# A high carbohydrate, but not fat or protein meal attenuates postprandial ghrelin, PYY and GLP-1 responses in Chinese men

**DOI:** 10.1371/journal.pone.0191609

**Published:** 2018-01-31

**Authors:** Ehsan Parvaresh Rizi, Tze Ping Loh, Sonia Baig, Vanna Chhay, Shiqi Huang, Jonathan Caleb Quek, E. Shyong Tai, Sue-Anne Toh, Chin Meng Khoo

**Affiliations:** 1 Department of Medicine, Yong Loo Lin School of Medicine, National University of Singapore, Singapore; 2 Department of Medicine, National University Health System, Singapore; 3 Department of Laboratory Medicine, National University Health System, Singapore; 4 Food Science and Technology Program, Department of Chemistry, Faculty of Science, National University of Singapore, Singapore; 5 Duke-National University of Singapore Medical School, Singapore; 6 Perelman School of Medicine, University of Pennsylvania, Philadelphia, Pennsylvania, United States of America; National Institute for Agronomic Research, FRANCE

## Abstract

It is known that the macronutrient content of a meal has different impacts on the postprandial satiety and appetite hormonal responses. Whether obesity interacts with such nutrient-dependent responses is not well characterized. We examined the postprandial appetite and satiety hormonal responses after a high-protein (HP), high-carbohydrate (HC), or high-fat (HF) mixed meal. This was a randomized cross-over study of 9 lean insulin-sensitive (mean±SEM HOMA-IR 0.83±0.10) and 9 obese insulin-resistant (HOMA-IR 4.34±0.41) young (age 21–40 years), normoglycaemic Chinese men. We measured fasting and postprandial plasma concentration of glucose, insulin, active glucagon-like peptide-1 (GLP-1), total peptide-YY (PYY), and acyl-ghrelin in response to HP, HF, or HC meals. Overall postprandial plasma insulin response was more robust in the lean compared to obese subjects. The postprandial GLP-1 response after HF or HP meal was higher than HC meal in both lean and obese subjects. In obese subjects, HF meal induced higher response in postprandial PYY compared to HC meal. HP and HF meals also suppressed ghrelin greater compared to HC meal in the obese than lean subjects. In conclusion, a high-protein or high-fat meal induces a more favorable postprandial satiety and appetite hormonal response than a high-carbohydrate meal in obese insulin-resistant subjects.

## Introduction

Obesity is a state of excess calorie intake and appetite dysregulation [1]. Satiety promoting diets that result in lower caloric intake may be helpful in promoting weight loss in obese individuals [2]. A low carbohydrate diet has been found to be an effective dietary regimen for weight loss [3, 4], and high protein diet has been suggested to be more satiating than other diet compositions [5].

Short-acting satiety hormonal signals consist of ghrelin, peptide YY (PYY), and glucagon like peptide-1 (GLP-1), which regulate calorie intake through their appetite-stimulating (orexigenic) or appetite-inhibiting (anorexigenic) effects [6]. PYY and GLP-1 are anorexigenic hormones, secreted from endocrine L-cells located in distal segment of jejunum and ileum [7, 8]. PYY acts on neuropeptide Y (NPY) Y2 receptors located in the arcuate nucleus [9], and GLP-1 through activation of pro-opiomelanocortin (POMC)/cocaine- and amphetamine-regulated transcript (CART) and inhibition of the NPY/agouti-related peptide (NPY/AgRP) neurons [10], to exert their inhibitory effects on appetite in humans. Ghrelin, a potent orexigenic hormone is secreted by endocrine cells in stomach, increases during the pre-prandial state leading to hunger and desire for food intake [11]. Ghrelin exerts its orexigenic effects on appetite centrally through an endogenous ligand for the growth hormone secretagogue receptor 1a (GHS-R1a) [12].

Emerging evidence suggests that nutrient intake regulates secretion of appetite hormones in both lean and obese individuals [13–26]. However, it is not clear which meal composition (high-carbohyrate, high-fat or high-protein) can promote and maintain greater satiety hormonal responses in people with obesity. Here, we aimed to determine the effects of isocaloric mixed meals with different macronutrient composition on postprandial PYY, GLP-1, and ghrelin responses between obese insulin-resistant and lean insulin-sensitive subjects over an extended duration (6 h) following a meal ingestion.

## Material and methods

This study was designed and conducted according to the Singapore Good Clinical Practice guideline, and principles of the 2013 Declaration of Helsinki. Singapore’s National Healthcare Group Domain Specific Review Board (DSRB Ref No: C/2013/00902) reviewed and approved the protocol of this study. All subjects provided written consent before participation in this study.

### Study subjects

We recruited nine obese (BMI ≥27.5 kg/m^2^) insulin-resistant and nine lean (BMI ≥18.5 and ≤23 kg/m^2^) insulin-sensitive Chinese men aged 21–40 years. We used BMI of 27.5 or higher to classify obesity in Asians, as Asians have higher risk of metabolic disease at lower BMI [27]. All subjects had fasting plasma glucose <5.6 mmol/l. We used the Homeostatic Model Assessment-Insulin Resistance (HOMA-IR) to classify our subjects as insulin-sensitive (HOMA-IR<1.2) and insulin-resistant (HOMA-IR≥2.5) [28]. Exclusion criteria included known first-degree family history or history of diabetes mellitus, current thyroid disorders, history of malignancy, hospitalization or surgery during the past 6 months, treatment for dyslipidemia, use of any medication during the past three months, daily alcohol consumption >3 units, and high level of physical activity (>5 hour per week). None of the subjects had ≥5% changes in their weight within the past three months before the study.

### Study protocol

Eligible subjects were subjected to three randomly assigned liquid mixed meal challenge tests, which were performed seven days apart. We have reported that the fasting plasma glucose and insulin concentrations within- (CVi) and inter-individual variation (CVg) were not different between lean and obese subjects between the three study visits [29]. Fasting and postprandial (30, 60, 90, 120, 180, and 360 min.) venous blood samples were collected for the measurement of plasma active GLP-1, total PYY, and acyl-ghrelin concentrations. Blood samples were collected into plastic tubes containing EDTA Na_2_ (VACUETTE^®^, Greiner Bio-One, Austria), 0.5mg DPP-4 inhibitor (Sigma-Aldrich, Darmstadt, Germany), and half a tablet of EDTA-free protease inhibitor (Roche, Mannheim, Germany). All blood samples were well mixed and chilled in an ice bath until centrifugation at 3000×g; 15min at 4°C; plasma was immediately separated and stored at -80°C until analysis.

For each meal challenge test, subjects underwent a 10-hour overnight fast. An isocaloric (≈600 kcal) isovolumic (≈400 ml) liquid meal was then given to the subjects to be ingested within 5 minutes. The three meal challenge tests were high-protein (HP), high-fat (HF), and high-carbohydrate (HC) meals which comprised of 51.4% energy from protein, 56.5% energy from fat (with a 1:1:1 ratio of saturated, mono- and poly-unsaturated fatty acids), and 56.4% energy from carbohydrate, respectively (S1 Table).

### Biochemical analysis

Plasma glucose and insulin were measured using an enzymatic method (AU5800, Beckman Coulter Inc., CA, USA) and a chemiluminescence immunoassay (ADVIA Centaur, Siemens Healthcare Diagnostics, Hamburg, Germany), respectively. For appetite hormones analysis, plasma concentration of active GLP-1, total PYY, and acyl-ghrelin was determined using the high-sensitivity enzyme-linked immunosorbent assay (ELISA) kits from Millipore, Billerica, MA, USA (Cat no. EZGLPHS-35K, EZHPYYT66K, and EZGRA-88K, respectively). All samples were assayed in duplicate in a single laboratory by the same technician. The intra- and inter-assay coefficient of variation (CV) were 9.0% and 5.2% for GLP-1, 4.4% and 8.9% for total PYY, 5.7% and 9.6% for ghrelin.

### Statistical analysis

All statistical analyses were performed using SPSS version 23.0 for Windows (SPSS Inc., Chicago, IL, USA). All values are presented as means±standard errors (SEMs). A *P* value of <0.05 was considered statistically significant.

HOMA-IR was calculated using the following formula: fasting insulin (mU/l) × fasting glucose (mmol/l)/22.5. Incremental area under the curve (iAUC) for the postprandial hormonal response was calculated using trapezoid method. Initial power analysis was based on the postprandial ghrelin suppression; a sample size of nine subjects per group per test meal had at least 87% power at 5.0% significance level to show an average 64% decrease in postprandial ghrelin concentration [30].

Student’s t*-*test was used to test the continuous variables between groups. The differences in the iAUC values between groups with different meals were analysed by ANOVA. Because of the inter-individual variations in fasting levels of metabolic and appetite hormones, we calculated the percentage change from baseline at each time point for the postprandial hormonal responses ((value at time point/value at fasting)×100)-100). For ghrelin, four fasting samples before HC or HF meals had no detectable ghrelin (that is, below the 15 pg/mL lowest detectable level of ghrelin) and no response to the meals could be calculated for these individuals. Two lean subjects had no detectable PYY (i.e., below the lowest detectable level of the 6.5 pg/mL) at numerous time points. These participants were therefore excluded from the analysis of these particular hormones but were included in other analyses in which they had detected values.

The time course of each postprandial hormone response was analysed using a linear mixed model. Linear mixed model takes into account the correlation between the variables which provides a useful approach for analyzing repeated measurements [31]. Time and type of meal was entered as repeated measures; group, type of meal, and time as main effects followed by a Fischer’s LSD post hoc test. Differences of postprandial response between meals as well as groups were assessed using the time × meal, time × group, and meal × group interaction tests.

## Results

Obese subjects were older and had greater BMI and waist circumference. The HOMA-IR in the obese subjects were about four times higher than the lean subjects (Table 1). Fasting plasma glucose was similar between groups, but fasting plasma insulin was higher in obese subjects. Obese subjects had higher fasting GLP-1, and PYY, but lower ghrelin concentrations compared to lean subjects, however none of these differences reached statistical significance.

**Table 1:** Baseline anthropometry and fasting plasma biochemistry of study subjects.

	Lean	Obese	*P*
Age, *years*	23.2 ± 0.2	28.6 ± 1.4	**0.002**
BMI, *kg*.*m*^*-2*^	22.0 ± 0.2	30.1 ± 0.7	**<0.001**
Waist Circumference, *cm*	79.9 ± 0.5	100.8 ± 1.0	**<0.001**
Glucose, *mmol/l*	4.33 ± 0.05	4.68 ± 0.12	0.459
Insulin, *mU/l*	4.31 ± 0.52	21.04 ± 2.27	**<0.001**
HOMA-IR	0.83 ± 0.10	4.34 ± 0.41	**<0.001**
PYY, *pg/ml*	135.5±25.2	171.4±11.6	0.620
Ghrelin, *pg/ml*	313.3±60.8	188.8±30.9	0.226
GLP-1, *pM*	3.17±0.34	4.61±0.65	0.537

Values are means ± SEM. *P* value is adjusted for age. n = 9 for each group.

### Postprandial glucose and insulin responses

#### Glucose

**Fig 1:**
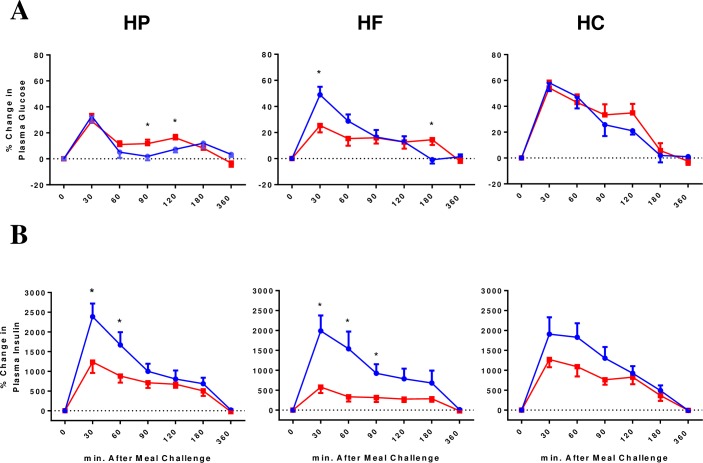
**Percentage change from baseline for plasma (A) glucose, and (B) insulin over 6 hours following ingestion of isocaloric and isovolumic high-protein (HP), high-fat (HF), or high-carbohydrate (HC) liquid mixed meals between lean insulin-sensitive (Blue, ●) and obese insulin-resistant (Red, ■) subjects (9 subjects in each group).** *P<0.05 for differences between lean and obese in plasma levels at the indicated meal challenge time point.

There were no statistical differences in the iAUC for plasma glucose between lean and obese subjects for all three meals (Table 2). The iAUC for plasma glucose was greater after HC meal compared to HP meal (*P* = 0.003) in lean subjects (Fig 1A). The iAUC for plasma glucose was greater after HC meal compared to HP (*P*<0.001) or HF meal (*P*<0.001) in obese subjects (Fig 1A).

**Table 2:** Incremental area-under-the-curve (iAUC) over 6 hours for plasma glucose, insulin, PYY, GLP-1, and ghrelin after ingestion of high-protein (HP), high-fat (HF) and high-carbohydrate (HC) meal.

		HP Meal	*P*	HF Meal	*P*	HC Meal	*P*
Glucose iAUC_0-360 min_ (*mmol*. *min*. *L*^*-1*^)	Obese	141.7 ± 15.6	0.824	147.0 ± 9.4	0.685	256.8 ± 39.7	0.376
	Lean	134.3 ± 28.4		161.0 ± 32.5		207.2 ± 37.4	
Insulin iAUC_0-360 min_ (*mU*. *min*. *L*^*-1*^)	Obese	26179.8 ± 5508.6	**0.011**	10508.0 ± 1846.0	**0.019**	20540.0 ± 2575.1	**0.001**
	Lean	9683.2 ± 1604.6		5378.3 ± 702.9		8374.5 ± 867.2	
PYY iAUC_0-360 min_ (*pg*.*min*.*ml* ^*-1*^)	Obese	25578.0±6714.2	0.431	20021.2±6365.2	0.315	13813.7±8981.3	0.939
	Lean	18957.8±4059.3		11074.1±4136.5		12971.9±3764.6	
GLP-1 iAUC_0-360 min_ (*pM*.*min*)	Obese	1285.6±250.9	0.914	1002.0±250.9	0.916	917.0±123.4	0.839
	Lean	1317.7±149.0		1040.7±259.0		974.2±248.0	
Ghrelin iAUC_0-360 min_ (*pg*.*min*.*ml* ^*-1*^)	Obese	-36041.8±9510.8	0.245	-24763.2±4420.5	0.386	-12774.8±17121.3	0.889
	Lean	-9552.8±15052.6		-10459.0±15283.9		-15745.3±12713.2	

Data are presented as the mean± SEM; n = 9 for each group. *P* value based on the repeated measure ANOVA between lean and obese subjects.

#### Insulin

The iAUC for plasma insulin was significantly higher in obese compared to lean subjects after all three meals. Among obese subjects, the iAUC for plasma insulin was higher after HP (*P* = 0.005) and HC (*P* = 0.002) meals compared to HF meal. There was no significant difference in the iAUC for plasma insulin between meals in lean subjects (Table 2).

Lean individuals had lower fasting insulin and as such, the percentage change in plasma insulin was greater in lean compared to obese subjects after HP (*P* = 0.031) and HF (*P* = 0.010) meal, but not HC meal (*P* = 0.100) (Fig 1B).

### Postprandial ghrelin, PYY, and GLP-1 responses

The postprandial iAUC of ghrelin, PYY, and GLP-1 was not statistically different between lean and obese subjects for all three meals (Table 2).

**Fig 2:**
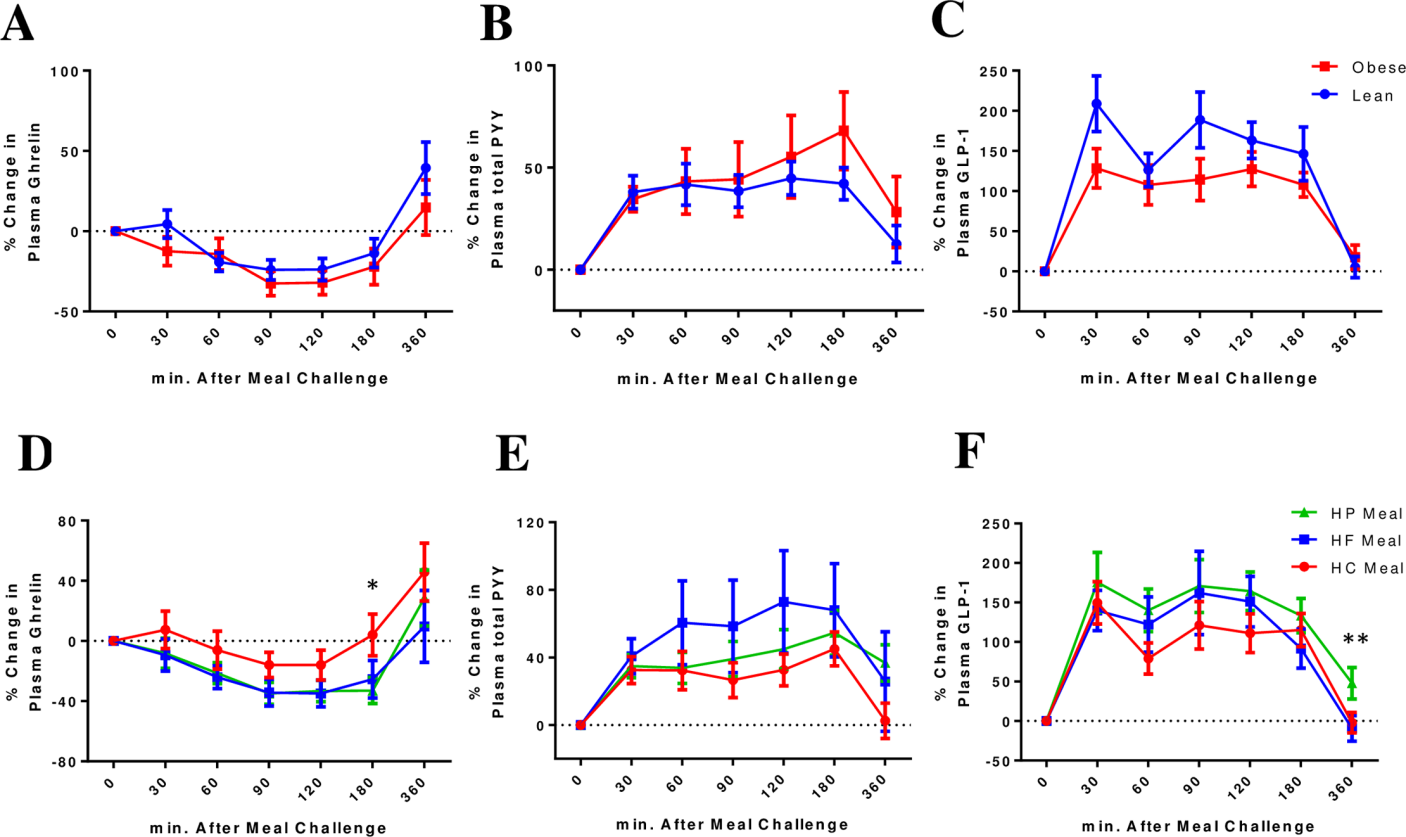
**Percentage change from baseline for plasma ghrelin, PYY, and GLP-1 over 6 hours between lean insulin-sensitive (Blue, ●) and obese insulin-resistant (Red, ■) subjects (2A, 2B, and 2C) and following ingestion of isocaloric and isovolumic high-protein (HP), high-fat (HF), or high-carbohydrate (HC) liquid mixed meals (2D, 2E, and 2F).** *P<0.05 for difference between HP vs. HC meal for ghrelin response. **P<0.05 for difference between HP vs. HC meal and HP vs. HF meal for GLP-1 response.

We used the linear mixed model to examine the main effect and interaction of lean or obese phenotype and type of meal on the postprandial response of ghrelin, PYY and GLP-1. The overall mean postprandial responses were not statistically different between lean and obese subjects for ghrelin, PYY and GLP-1 (Table 3). HF meal induced significantly greater postprandial hormone responses compared to HC meal (greater suppression in ghrelin and higher responses in PYY and GLP-1). HP meal induced lower postprandial responses in ghrelin and higher postprandial responses in GLP-1 compared to HC meal. The plasma level of GLP-1 was significantly higher after HP meal compared to HF or HC meal at 6-hour after the meal intake. (Fig 2F).

**Table 3:** Percentage change in plasma ghrelin, PYY, and GLP-1 over 6-hour after high-protein (HP), high-fat (HF), or high-carbohydrate (HC) meal using linear mixed models.

	Ghrelin	PYY	GLP-1
**Lean**	-5.5 (-18.6–7.7)	31.6 (12.5–50.7)	119.8 (77.2–162.4)
**Obese**	-15.6 (-28.9 –-2.2)	39.2 (21.6–56.8)	86.2 (43.5–128.8)
**HP Meal**	-15.6 (-26.3 –-4.9) ^a^	36.2 (20.6–51.7)	118.9 (85.9–151.8) ^a^
**HF Meal**	-20.3 (-31.4 –-9.2) ^b^	45.1 (29.8–60.3) ^**b**^	108.1 (75.1–141.0) ^b^
**HC Meal**	4.4 (-6.4–15.1) ^a^^,^^b^	25.0 (9.5–40.5) ^**b**^	82.0 (49.1–114.9) ^a^^,^^b^
***P* for Meal Effect**	**0.001**	**0.037**	**0.011**
***P* for Group Effect**	0.273	0.548	0.257
***P* for Time Effect**	**0.001**	**0.001**	**0.001**
***P* interaction for Group × Time**	0.597	0.861	0.129
***P* interaction for Meal × Time**	0.779	0.960	0.931
***P* interaction for Meal × Group**	**0.001**	**0.011**	0.245

Data presented as Mean (95% CI).

^a^*P* <0.05 for HP vs HC

^b^*P* <0.05 for HF vs HC.

Next, we examined whether the lean or obese phenotype modulates the effect of different meals on the postprandial responses in ghrelin, PYY and GLP-1. The postprandial trajectories for ghrelin tracked significantly lower after HP or HF meal but higher after HC meal among obese subjects but not lean subjects (*P* interaction meal × group<0.001) (Table 3 and S2A Fig). Among obese subjects, the postprandial trajectories for PYY tracked significantly higher after HF meal, but not after HP meal, HC meal or among lean subjects (*P* inteaction meal × group = 0.011) (Table 3 and S2B Fig). The HP or HF meal induced a higher postprandial GLP-1 response compared to HC meal, regardless of lean or obese phenotype (*P* interaction meal **×** group>0.05).

## Discussions

Several studies have demonstrated changes in the gut hormonal profile in response to an acute meal challenge with different macronutrient composition in humans, but mostly within a short duration (i.e. 2 to 4 hours) after the meal. Here, we studied whether gut hormonal profile differs between obese and lean subjects in response to an acute meal challenge using a liquid meal rich in protein, fat, or carbohydrate over an extended postprandial duration of up to 6 hours, consistent with the human’s habitual food intake. We also deliberately selected obese insulin-resistant individuals, and compared their postprandial hormonal responses to lean insulin-sensitive individuals. We showed that in the obese subjects, a meal rich in carbohydrate resulted in a smaller increment in plasma GLP-1 and PYY and less suppression of ghrelin concentration over 6 hours after meal intake compared to meals rich in fat or protein. Among obese insulin-resistant subjects, we also observed a greater insulin response following a high-protein or high-carbohydrate meal compared to a high-fat meal.

In lean subjects, the postprandial ghrelin suppression was similar after all three meals (S2A Fig), which agrees with an earlier study in non-obese individuals [25]. However, two other studies in lean and overweight individuals reported that a high-protein meal induces greater ghrelin suppression compared to a high-fat or high-carbohydrate meal throughout the 3-h [32] and 6-h post-meal period [19]. We showed that in the obese subjects, a high-fat or high-protein meal was more effective than a high-carbohydrate meal in postprandial ghrelin suppression (S2A Fig). Indeed, previous studies have suggested that a high-protein meal reduces appetite, and extends the food-free interval between meals [33, 34], potentially through a greater postprandial ghrelin suppression [35]. A post-gastric mechanism has been proposed to underlie the postprandial ghrelin suppression. Faster gastric transition and post-gastric absorption of carbohydrates lead to a more rapid but shorter duration of ghrelin suppression. Conversely, a longer gastric transition time with high fat or protein in the diet might lead to a longer duration of ghrelin suppression [36].

GLP-1 and PYY are two gut hormones, secreted together after a meal, to provide a short-term and intermediate-term “brake” signal on the food intake. Coinfusion of GLP-1 and PYY, in healthy overweight men, is associated with reduced food intake [37]. After bariatric surgery, there is a marked elevation in the postprandial plasma GLP-1 and PYY, and this has been shown to reduce postoperative *ad libitum* food intake [38]. In our study, a high-carbohydrate meal induced lower postprandial GLP-1 and PYY responses compared to a high-fat meal. Gibbons and colleagues reported that a high-fat meal induces greater rise in the postprandial GLP-1 and PYY responses than a high-carbohydrate meal in 16 overweight and obese individuals, which is associated with a higher degree in late satiety [26]. Our findings agree with this study, although we did not have the subjective assessment for satiety sensations. Other investigators have shown an increased plasma PYY and GLP-1 after a high-protein meal (containing casein and whey protein) in lean, healthy subjects at 4 hours after the meal [25]. We add to the evidence by showing the stimulatory effect of a high-protein meal on postprandial plasma PYY and GLP-1 over an extended 6 hours post-meal in obese insulin-resistant individuals. Direct stimulation of enteroendocrine cells by amino acids has been proposed as a trigger factor for the PYY and GLP-1 secretion. The calcium-sensing receptors (CaSR) have been shown to act as an l-amino acid sensor in the L-cells. Therefore, activation of the CaSR following exposure to a wide range of amino acids in the diet can lead to GLP-1 and PYY secretion from the gut [39]. Moreover, glutamine has been shown to stimulate GLP-1 secretion through increasing calcium and cAMP in ex-vivo L-cells [40].

All our subjects had normal glucose tolerance; however, the obese insulin-resistant subjects had fasting hyperinsulinemia, but significantly lower percentage change in insulin response compared to lean insulin-sensitive subjects. Kahn et al. [41] described a hyperbolic relationship between insulin sensitivity and β-cell function in healthy human subjects with normal glucose tolerance within a wide range of body mass index. Our findings of lower postprandial insulin response would be compatible with an early β-cell dysfunction in insulin resistant subjects. The postprandial GLP-1 response between lean and obese was similar after the high-protein and high-carbohydrate meal, indicating that the incretin axis was not aberrant in obese insulin-resistant subjects. We also showed that among obese insulin-resistant subjects, a high-protein or high-carbohydrate meal induces higher postprandial insulin response compared to a high-fat meal. Amino acids have been shown to stimulate insulin secretion from pancreatic β-cells in both *in vivo* and *in vitro* studies [42]. Whey protein which was the source of protein in our study has been shown to have an insulinogenic effect with only mild changes in glycaemia in healthy subjects [43].

Our study has several important advantages. Our findings add to the current literature on the postprandial appetite hormonal response at a longer duration after an acute meal challenge [to mimic duration of habitual eating in humans]. We provided hormonal assessments in response to meals rich in three major macronutrients of similar calorie and texture. Our cohorts had very distinct metabolic phenotypes being lean insulin-sensitive and obese insulin-resistant. It is important to note that not all obese individuals are insulin resistant and not all lean individuals are insulin sensitive. There are several limitations to this study. We used an isocaloric and isovolumic liquid-mixed meal with various macronutrient contents to test our hypothesis. Gastric emptying is more prolonged after a solid meal compared to a liquid meal [44] which might affect peptide release from enterocytes consequently. However, in a pilot study, liquid-mixed meal showed to be better and more uniformly tolerated among the study subjects, with a greater stimulated incretin and insulin response compared to a solid-mixed meal [45]. The study was limited to a small sample size despite a very well characterized cohort of lean and obese individuals. However, previous studies on the effect of different diet composition on satiety and appetite hormones secretion have been performed with comparable sample size [15, 18–21, 23–25]. Moreover, undetectable fasting and postprandial plasma PYY in 2 lean subjects, might have decreased the power to reject the null hypothesis for any difference between lean and obese subjects. We also limited our study subjects to Chinese males, and future study will be required to validate these results in a larger cohort consisting of other ethnic groups and to include females. A previous meal might have an effect on the secretion of gut hormones, for example fermentable carbohydrates induces a higher endogenous GLP-1 and PYY secretion 10 hours after the meal intake [46]. Nonetheless, we asked all subjects to have a light snack the night before, all subjects fasted for 10 hours before study procedures and we did not find statistical differences in the fasting plasma ghrelin, PYY and GLP-1 concentrations. Lastly, appetite regulation is a complex process, and concurrent assessment of psychometric hunger scores and functional brain imaging of appetite regulating regions might give us a more reassuring assessment of effects of macronutrient composition on appetite regulation.

In conclusion, we showed that a high-protein or high-fat meal induces greater postprandial GLP-1, PYY and ghrelin responses compared to a high-carbohydrate meal over a 6-hour post meal. The effects of macronutrients on gut hormonal response are more pronounced in the obese than lean subjects. Increasing protein and fat content while minimizing carbohydrate in the meal could be used as a dietary strategy to promote longer satiation among obese insulin-resistant individuals.

## Supporting information

S1 Table Macronutrient composition of the 3 different liquid mixed meals. HC, high carbohydrate, HF, high fat, HP, high protein, MUFA, monounsaturated fatty acids, PUFA, polyunsaturated fatty acids, SFA, saturated fatty acids, Ensure Plus^®^ (1g = 1.41kcal, 0.05g protein, 0.045g fat, 0.1988g carbohydrate, 0.0057g SFA, 0.01095g MUFA, 0.02655g PUFA, 0g fibre) manufactured by Abbott Nutrition was used as a benchmark for HC meal; Beneprotein^®^ (1g powder = 3.57kcal, 0g fat, 0g carbohydrate, 0.85g protein, 5mg potassium, 5.7mg calcium, 2mg phosphorus, 0g fibre) is manufactured by Nestlé Nutrition.
(PDF)

S1 Fig **Postprandial plasma (A) PYY, *pg/ml*; (B) GLP-1, *pM*, and (C) ghrelin, *pg/ml*; in 9 lean insulin-sensitive (Blue, ●), and 9 obese insulin-resistant (Red, ■) subjects over 6 hours following ingestion of isocaloric and isovolumic high protein (HP), high fat (HF), or high carbohydrate (HC) liquid mixed meals.** *P<0.05 for difference between lean vs. obese subjects.
(PDF)

S2 Fig **Percentage change from baseline for plasma (A) ghrelin and (B) PYY in 9 lean insulin-sensitive and 9 obese insulin-resistant subjects over 6 hours following ingestion of 3 different liquid mixed meals.** HP, high-protein; HF, high-fat; HC, high-carbohydrate.
(PDF)
