# Supplementary material for: A high carbohydrate, but not fat or protein meal attenuates postprandial ghrelin, PYY and GLP-1 responses in Chinese men
Source: PLoS One. 2018 Jan 31;13(1):e0191609. doi: 10.1371/journal.pone.0191609 (PMC5792004; doi:10.1371/journal.pone.0191609)
Supplement: S1 Table — HC, high carbohydrate, HF, high fat, HP, high protein, MUFA, monounsaturated fatty acids, PUFA, polyunsaturated fatty acids, SFA, saturated fatty acids, Ensure Plus® (1g = 1.41kcal, 0.05g protein, 0.045g fat, 0.1988g carbohydrate, 0.0057g SFA, 0.01095g MUFA, 0.02655g PUFA, 0g fibre) manufactured by Abbott Nutrition was used as a benchmark for HC meal; Beneprotein® (1g powder = 3.57kcal, 0g fat, 0g carbohydrate, 0.85g protein, 5mg potassium, 5.7mg calcium, 2mg phosphorus, 0g fibre) is manufactured by Nestlé Nutrition. (PDF) [file pone.0191609.s001.pdf]

S1 Table. Macronutrient composition of the 3 different liquid mixed meals

		Amount (g)	Energy (Kcal)	Carb. (g)	Protein (g)	Fat (g)	MUFA (g)	PUFA (g)	SFA (g)
**HF Meal**	*Ensure Plus*®	260.0	366.60	51.69	13.00	11.70	2.85	6.90	1.48
	Butter	11.0	78.87	0.01	0.09	8.80	2.31	0.33	5.61
	Diary Cream Heavy Whipping	11.0	37.95	0.31	0.22	4.07	1.10	0.15	2.53
	Peanut Oil	13.0	114.92	0.00	0.00	13.00	5.98	4.16	2.18
	Total		**598.34**	52.00	13.31	37.57	12.24	11.54	11.81
	% composition						32.6	30.7	31.4
	% Kcal			34.8	8.9	**56.5**			
**HC Meal**	*Ensure Plus*®	425.0	599.25	84.49	21.25	19.13	4.65	11.28	2.42
	Total		**599.25**	84.49	21.25	19.13	4.65	11.28	2.42
	% composition						24.3	59.0	12.7
	% Kcal			**56.4**	14.2	28.7			
**HP Meal**	*Ensure Plus*®	230.0	324.30	45.72	11.50	10.35	2.52	6.11	1.31
	Beneprotein® Powder	77.0	274.89	0.00	65.45	0.00	0.00	0.00	0.00
	Total		**599.19**	45.72	76.95	10.35	2.52	6.11	1.31
	% composition						24.3	59.0	12.7
	% Kcal			30.5	**51.4**	15.5			

HC, high carbohydrate, HF, high fat, HP, high protein, MUFA, monounsaturated fatty acids, PUFA, polyunsaturated fatty acids, SFA, saturated fatty acids, *Ensure Plus*® (1g=1.41kcal, 0.05g protein, 0.045g fat, 0.1988g carbohydrate, 0.0057g SFA, 0.01095g MUFA, 0.02655g PUFA, 0g fibre) manufactured by *Abbott Nutrition* was used as a benchmark for HC meal; *Beneprotein*® (1g powder=3.57kcal, 0g fat, 0g carbohydrate, 0.85g protein, 5mg potassium, 5.7mg calcium, 2mg phosphorus, 0g fibre) is manufactured by *Nestlé Nutrition*.
