# Supplementary material for: A high carbohydrate, but not fat or protein meal attenuates postprandial ghrelin, PYY and GLP-1 responses in Chinese men
Source: PLoS One. 2018 Jan 31;13(1):e0191609. doi: 10.1371/journal.pone.0191609 (PMC5792004; doi:10.1371/journal.pone.0191609)
Supplement: S1 Fig — Postprandial plasma (A) PYY, pg/ml; (B) GLP-1, pM, and (C) ghrelin, pg/ml; in 9 lean insulin-sensitive (Blue, ●), and 9 obese insulin-resistant (Red, ■) subjects over 6 hours following ingestion of isocaloric and isovolumic high protein (HP), high fat (HF), or high carbohydrate (HC) liquid mixed meals. *P<0.05 for difference between lean vs. obese subjects. (PDF) [file pone.0191609.s002.pdf]

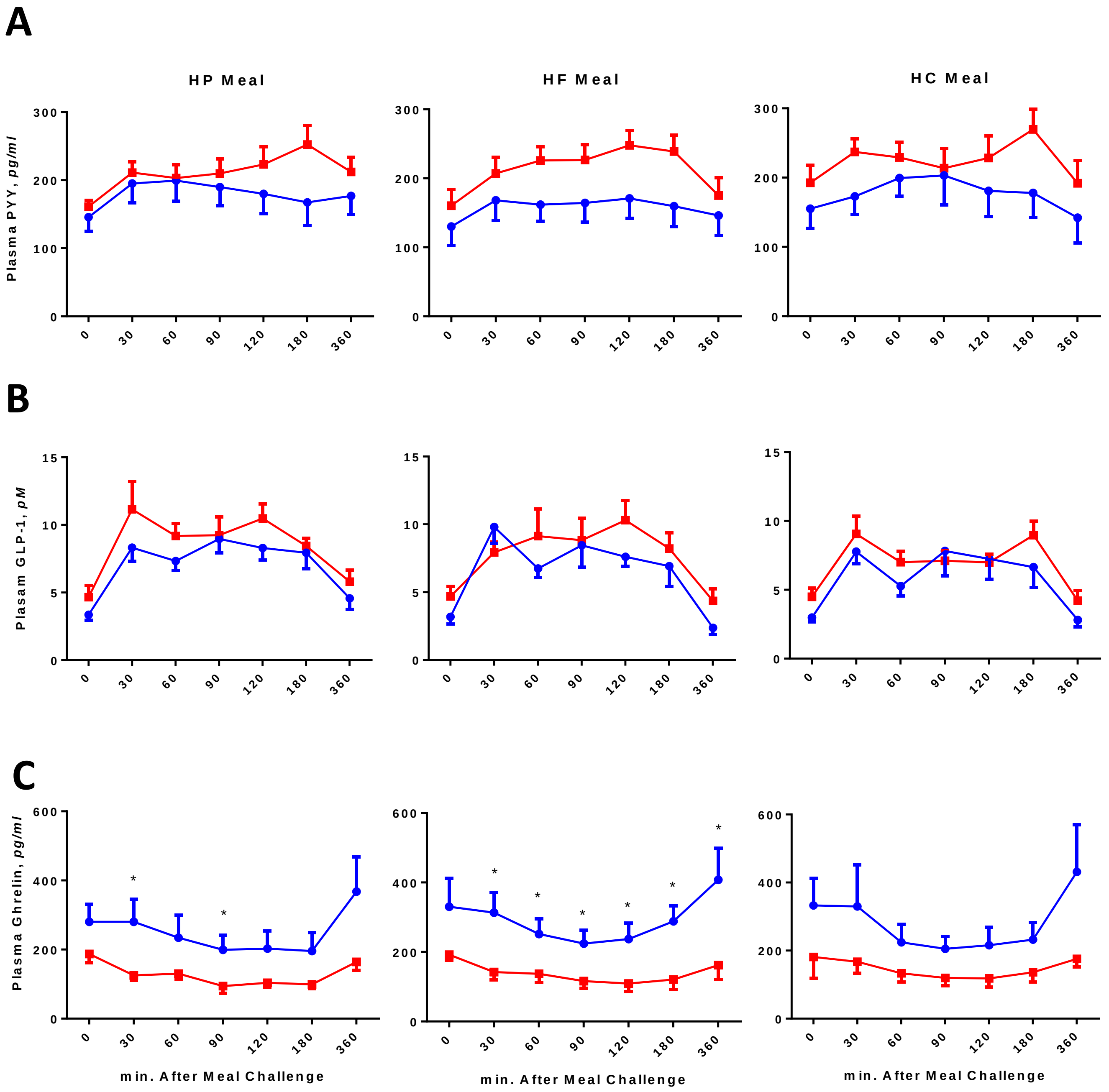

S1 Fig. Postprandial plasma (A) PYY, *pg/ml*; (B) GLP-1, *pM*, and (C) ghrelin, *pg/ml*; in 9 lean insulin-sensitive (—, ●), and 9 obese insulin-resistant (—, ■) subjects over 6 hours following ingestion of isocaloric and isovolumic high-protein (HP), high-fat (HF), or high-carbohydrate (HC) liquid mixed meals. * P<0.05 for difference between lean vs. obese subjects.
