# Supplementary material for: A high carbohydrate, but not fat or protein meal attenuates postprandial ghrelin, PYY and GLP-1 responses in Chinese men
Source: PLoS One. 2018 Jan 31;13(1):e0191609. doi: 10.1371/journal.pone.0191609 (PMC5792004; doi:10.1371/journal.pone.0191609)
Supplement: S2 Fig — Percentage change from baseline for plasma (A) ghrelin and (B) PYY in 9 lean insulin-sensitive and 9 obese insulin-resistant subjects over 6 hours following ingestion of 3 different liquid mixed meals. HP, high-protein; HF, high-fat; HC, high-carbohydrate. (PDF) [file pone.0191609.s003.pdf]

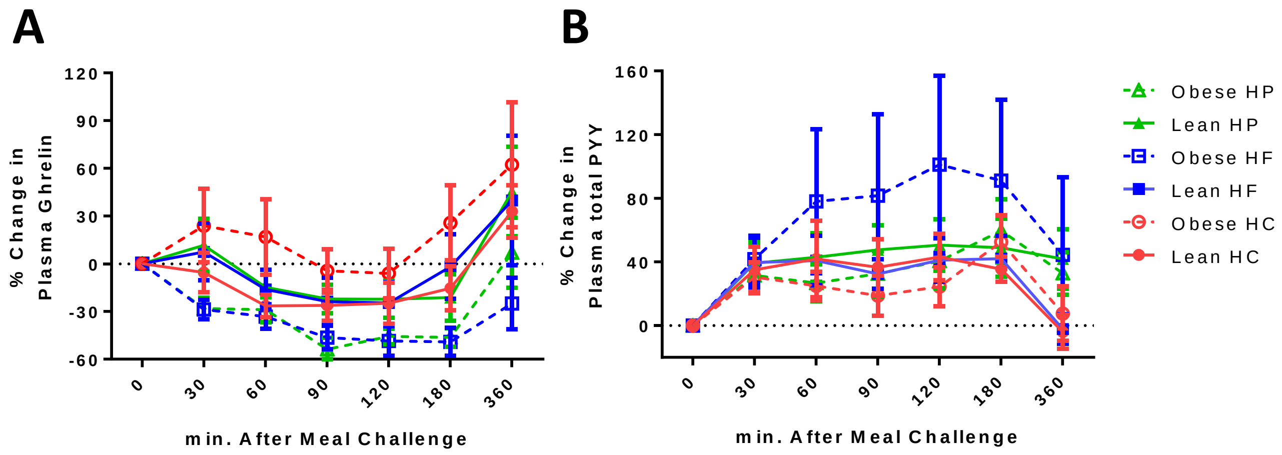

S2 Fig. Percentage change from baseline for plasma (A) ghrelin; and (B) PYY; in 9 lean insulin-sensitive and 9 obese insulin-resistant subjects over 6 hours following ingestion of 3 different liquid mixed meals. HP, high-protein; HF, high-fat; HC, high-carbohydrate.
